# Lupus Panniculitis of the Buttock: A Refractory Case With Diagnostic Challenges

**DOI:** 10.7759/cureus.115942

**Published:** 2026-09-08

**Authors:** Lamis El Yamani, Ouissal Hormi, Nassiba Zerrouki, Nada Zizi

**Affiliations:** 1 Dermatology, Allergology and Venerology, Centre Hospitalo-Universitaire Mohammed VI, Oujda, MAR

**Keywords:** chronic cutaneous lupus erythematosus, lupus erythematosus panniculitis, lupus profundus, methotrexate, ulcerative lupus panniculitis

## Abstract

Lupus erythematosus panniculitis (LEP) is a rare form of chronic cutaneous lupus involving the subcutaneous fat. Ulcerative lesions are uncommon and may mimic infectious, inflammatory, or neoplastic disorders, leading to delayed diagnosis. We report the case of a 40-year-old woman with longstanding photosensitivity who presented with a painful left gluteal ulcer evolving over eight months. The lesion began as a deep inflammatory nodule and progressively ulcerated. Antibiotic therapy was ineffective. Laboratory tests showed positive antinuclear antibodies at 1:160 and positive anti-Sm/RNP antibodies, with negative anti-double-stranded DNA antibodies. Histopathology revealed lymphocytic lobular panniculitis consistent with lupus panniculitis. Treatment with hydroxychloroquine and systemic corticosteroids resulted in partial improvement, but relapses occurred during steroid tapering. Dapsone failed to control the disease. Methotrexate was subsequently introduced, leading to marked clinical improvement within three months. This case highlights an unusual ulcerative gluteal presentation of lupus panniculitis. Early biopsy and clinicopathological correlation are essential to exclude mimickers and guide appropriate immunomodulatory treatment.

## Introduction

Lupus erythematosus panniculitis (LEP), also known as lupus profundus, is a rare form of chronic cutaneous lupus erythematosus characterized by inflammation of the deep dermis and subcutaneous adipose tissue [1,2]. It accounts for approximately 2%-5% of cases of cutaneous lupus erythematosus and predominantly affects women [1,3]. Clinically, LEP usually presents as firm, painful subcutaneous nodules or infiltrated plaques, most commonly involving the face, proximal upper limbs, trunk, breasts, thighs, and buttocks [2,3]. Healing may lead to permanent lipoatrophy, depressed scars, and contour deformities [1,3].

The diagnosis is often challenging because LEP may clinically mimic infectious, inflammatory, vascular, or neoplastic disorders [2,4]. Infectious panniculitis, including bacterial, mycobacterial, and deep fungal infections, should be considered particularly in ulcerative presentations. Subcutaneous panniculitis-like T-cell lymphoma represents an important neoplastic differential diagnosis because of its overlapping clinical and histopathological features with LEP [4,5]. Ulceration is an uncommon manifestation of LEP, and presentations characterized by an isolated ulcerative lesion confined to the gluteal region are particularly unusual [3,4]. These atypical features may make clinical recognition more difficult and contribute to delayed diagnosis. Histopathological examination remains essential to confirm the diagnosis and to exclude important mimickers, especially subcutaneous panniculitis-like T-cell lymphoma [4,5]. Antimalarial therapy is generally considered the first-line treatment for LEP, whereas systemic corticosteroids and other immunosuppressive agents may be considered for more extensive or treatment-resistant disease [1,2]. Although disease control can usually be achieved with appropriate therapy, residual lipoatrophy and permanent scarring may persist after resolution of the inflammatory lesions [1,2].

We report a rare case of ulcerative lupus panniculitis localized to the gluteal region in a 40-year-old woman with longstanding photosensitivity, highlighting an uncommon ulcerative presentation at an isolated gluteal site that contributed to a prolonged diagnostic delay.

## Case presentation

A 40-year-old woman with a history of photosensitivity presented with an eight-month history of a progressively enlarging and painful lesion of the left buttock. The condition initially appeared as a deep subcutaneous nodule that gradually evolved into an ulcerative lesion despite several courses of systemic antibiotics. Further history-taking revealed no other manifestations suggestive of systemic lupus erythematosus, including malar or other cutaneous eruptions, oral ulcers, alopecia, arthralgia or arthritis, Raynaud phenomenon, or symptoms suggestive of renal, neurological, cardiac, or pulmonary involvement. She reported no fever, night sweats, unintentional weight loss, or other constitutional symptoms suggestive of an infectious or lymphoproliferative process. Her medical history was otherwise unremarkable, with no known autoimmune disease or significant comorbidities.

At the time of dermatological evaluation, physical examination revealed an approximately 8 × 5 cm ulcer on the left gluteal region with a clean granulating erythematous base, focal fibrin deposits, and violaceous inflammatory borders. A small adjacent papulonodular satellite lesion was also present (Figure 1). Examination of the remaining skin and mucosal surfaces showed no additional lesions suggestive of cutaneous lupus. Given its painful ulcerative presentation, a broad differential diagnosis was considered, including infectious ulceration or panniculitis, deep fungal or mycobacterial infection, cutaneous vasculitis, subcutaneous panniculitis-like T-cell lymphoma, and ulcerative lupus panniculitis. Pyoderma gangrenosum was also considered; however, it remained a diagnosis of exclusion.

**Figure 1:**
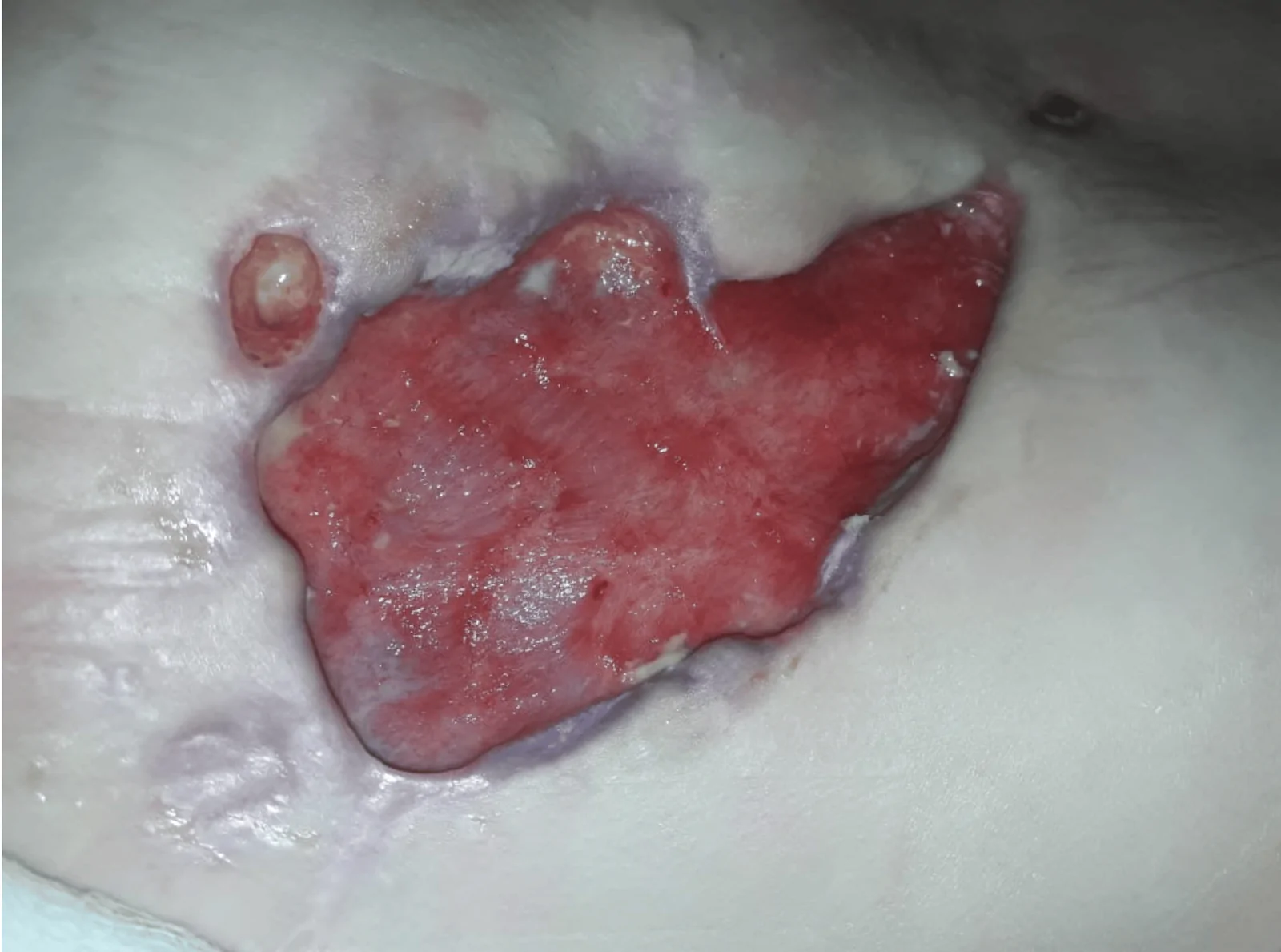
Ulcerative lupus panniculitis of the left gluteal region. A large, painful ulcer on the left buttock, displaying irregular geographic contours, a granulating base with scattered fibrinous areas, and undermined erythematous edges. The lesion evolved from a deep inflammatory subcutaneous nodule that progressively broke down, leading to marked local pain and tissue loss. This unusual ulcerative pattern made the diagnosis particularly challenging.

During the initial diagnostic work-up, general laboratory investigations showed normal hematological, renal, and hepatic parameters, with hemoglobin of 13.0 g/dL, a white blood cell count of 5,000/mm³, a platelet count of 260,000/mm³, serum creatinine of 0.6 mg/dL, aspartate aminotransferase of 12 U/L, and alanine aminotransferase of 14 U/L. Inflammatory markers were elevated, with a C-reactive protein level of 50 mg/L and an erythrocyte sedimentation rate of 45 mm/h. The general laboratory investigation profile is summarized in Table 1. Microbiological cultures were negative, making an infectious cause less likely.

**Table 1:** General investigation profile Routine laboratory investigations showed elevated inflammatory markers (CRP and ESR), while hematological, renal, and hepatic parameters remained within normal limits.

Investigation	Patient result	Reference range	Interpretation
Hemoglobin	13.0 g/dL	12.0–16.0 g/dL	Normal
White blood cell count	5,000/mm³	4,000–10,000/mm³	Normal
Platelet count	260,000/mm³	150,000–400,000/mm³	Normal
C-reactive protein (CRP)	50 mg/L	<5 mg/L	Elevated
Erythrocyte sedimentation rate (ESR)	45 mm/h	<20 mm/h	Elevated
Serum creatinine	0.6 mg/dL	0.5–1.1 mg/dL	Normal
Aspartate aminotransferase	12 U/L	10–35 U/L	Normal
Alanine aminotransferase	14 U/L	7–35 U/L	Normal

A first deep skin biopsy was subsequently performed and revealed predominantly lobular lymphocytic panniculitis with adipocyte necrosis. Because these findings raised concern for subcutaneous panniculitis-like T-cell lymphoma, a second deep biopsy with immunohistochemical analysis was performed during the diagnostic work-up. The repeat biopsy demonstrated lobular lymphocytic panniculitis associated with extensive hyaline fat necrosis, lymphoid aggregates with reactive germinal centers, plasma cell infiltrates, and focal dermal mucin deposition. Immunohistochemistry showed a mixed CD3-positive T-cell infiltrate without significant atypia. The lack of significant lymphocytic atypia, together with the presence of reactive lymphoid aggregates, plasma cells, and dermal mucin, favored lupus panniculitis over subcutaneous panniculitis-like T-cell lymphoma. T-cell clonality analysis was not performed because it was not available at our institution.

The subsequent autoimmune work-up revealed positive antinuclear antibodies at a titer of 1:160 and positive anti-Sm/RNP antibodies, whereas anti-double-stranded DNA antibodies were negative. Complement C3 and C4 levels were within normal limits. The specific laboratory and microbiological investigations are summarized in Table 2. Taken together, the clinical, microbiological, histopathological, immunohistochemical, and serological findings supported the diagnosis of LEP.

**Table 2:** Specific laboratory and microbiological investigations performed during the diagnostic workup. ANA and anti-Sm/RNP antibodies were positive, whereas anti-dsDNA antibodies were negative. Complement C3 and C4 levels were within normal limits, and microbiological cultures showed no evidence of infection.

Investigation	Patient result	Reference/expected result
Antinuclear antibodies (ANA)	Positive, 1:160	Negative
Anti-Sm/RNP antibodies	Positive	Negative
Anti-double-stranded DNA (anti-dsDNA) antibodies	Negative	Negative
Complement C3	Within normal limits	Laboratory reference range
Complement C4	Within normal limits	Laboratory reference range
Microbiological cultures	Negative	Negative

Following confirmation of the diagnosis, treatment with hydroxychloroquine (400 mg/day), oral prednisone (1 mg/kg/day), and strict photoprotection was initiated, resulting in partial improvement. Prednisone was maintained at the initial dose until ulcer healing was achieved and was then gradually tapered by approximately 15% every three weeks. During corticosteroid tapering, repeated relapses occurred. Dapsone (100 mg/day) was therefore added and continued for three months but failed to achieve sustained disease control.m

After three months of unsuccessful dapsone therapy, methotrexate (25 mg/week) was added to the ongoing corticosteroid therapy as a steroid-sparing agent because of the refractory and corticosteroid-dependent course. Marked clinical improvement was observed within three months of methotrexate initiation, with progressive healing of the ulcer and resolution of inflammation, as documented clinically and photographically (Figure 2). This improvement allowed further corticosteroid tapering. Serial lesion measurements and standardized pain scores were not available. Methotrexate was continued for a total of 12 months. At the one-year follow-up, clinical remission was maintained without recurrence, and no treatment-related adverse events were observed. Residual atrophic scarring persisted at the site of the lesion. The chronological sequence of clinical events, investigations, treatments, and outcomes is summarized in Table 3.

**Figure 2:**
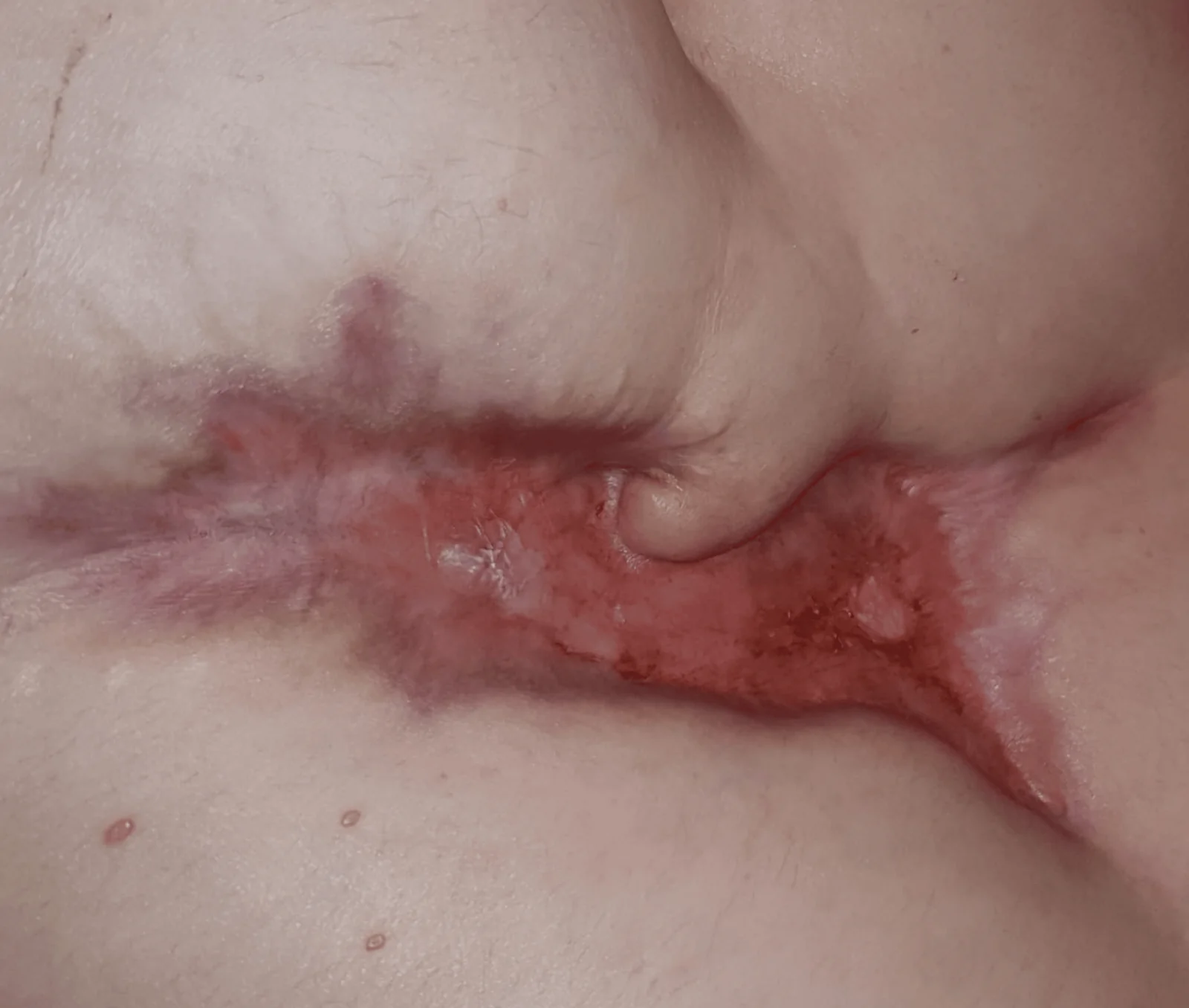
Clinical outcome after three months of treatment with hydroxychloroquine, corticosteroids, and methotrexate. At the three-month follow-up, a marked reduction in lesion size was observed, with extensive re-epithelialization and significant regression of the ulcerative process. The remaining lesion consisted mainly of shallow residual erosions surrounded by fibrotic and dyspigmented scar tissue. The absence of new ulceration and the progressive restoration of tissue integrity were consistent with a sustained therapeutic response.

**Table 3:** Timeline of clinical events and management ANA, antinuclear antibodies; anti-dsDNA, anti-double-stranded DNA antibodies; C3, complement component 3; C4, complement component 4.

Time point	Clinical event/investigation	Management and outcome
Eight months before presentation	Onset of a painful deep subcutaneous nodule on the left buttock	Progressive enlargement and subsequent ulceration despite several courses of systemic antibiotics
At dermatological presentation	Approximately 8 × 5 cm painful gluteal ulcer with an adjacent papulonodular lesion	Broad diagnostic work-up initiated
Initial diagnostic work-up	Elevated inflammatory markers; normal hematological, renal, and hepatic parameters; negative microbiological cultures	Infectious etiology considered less likely
Initial histopathological evaluation	First deep biopsy showed lobular lymphocytic panniculitis with adipocyte necrosis	Subcutaneous panniculitis-like T-cell lymphoma remained a concern
Subsequent diagnostic evaluation	Second deep biopsy and immunohistochemistry showed extensive hyaline fat necrosis, reactive lymphoid aggregates with germinal centers, plasma cell infiltrates, focal dermal mucin deposition, and no significant lymphocytic atypia	Findings favored lupus panniculitis; T-cell clonality analysis was unavailable
Autoimmune work-up	ANA 1:160 and anti-Sm/RNP positive; anti-dsDNA negative; C3 and C4 normal	Clinical, histopathological, immunohistochemical, and serological findings supported lupus panniculitis
After diagnosis	Hydroxychloroquine 400 mg/day, prednisone 1 mg/kg/day, and strict photoprotection initiated	Partial improvement and ulcer healing
During corticosteroid tapering	Prednisone gradually tapered by approximately 15% every three weeks	Repeated relapses occurred
Following relapses	Dapsone 100 mg/day added and continued for three months	No sustained disease control
After three months of dapsone	Methotrexate 25 mg/week added to ongoing corticosteroid therapy as a steroid-sparing agent	Treatment escalation because of refractory, corticosteroid-dependent disease
Three months after methotrexate initiation	Clinical and photographic reassessment	Marked ulcer healing and resolution of inflammation, allowing further corticosteroid tapering
Up to 12 months	Methotrexate continued	Clinical remission maintained without treatment-related adverse events
At the one-year follow-up	Clinical reassessment	No recurrence; residual atrophic scarring persisted

## Discussion

The diagnostic challenge in our patient was related to the combination of an isolated gluteal lesion, ulceration, and the absence of other clear manifestations of systemic lupus erythematosus. Although LEP may have a variable clinical presentation, atypical forms can mimic infectious, inflammatory, vascular, or neoplastic disorders and may therefore delay diagnosis [2,4]. In our patient, the chronic painful ulcerative appearance initially led to consideration of infectious panniculitis, deep fungal or mycobacterial infection, cutaneous vasculitis, pyoderma gangrenosum, and subcutaneous panniculitis-like T-cell lymphoma [4-6]. The negative microbiological investigations and the absence of systemic infectious features made an infectious etiology less likely.

Histopathological examination was essential in narrowing the diagnosis. The first biopsy demonstrated lobular lymphocytic panniculitis with adipocyte necrosis but raised concern for subcutaneous panniculitis-like T-cell lymphoma. A second deep biopsy therefore became necessary. The presence of extensive hyaline fat necrosis, reactive lymphoid aggregates with germinal centers, plasma cell infiltration, focal dermal mucin deposition, and the absence of significant cytological atypia favored lupus panniculitis [1,4,5]. Distinguishing lupus panniculitis from subcutaneous panniculitis-like T-cell lymphoma can be difficult because of overlapping clinical and histopathological findings, and repeated deep sampling with immunohistochemical evaluation may be required in equivocal cases [4,5]. T-cell clonality analysis may provide additional support in this setting, although it was not available at our institution.

The serological findings also supported a lupus-related process. Antinuclear antibody positivity is frequently reported in lupus panniculitis, whereas more specific autoantibodies vary according to the presence or absence of associated systemic disease [2,3]. In our patient, positive antinuclear antibodies and anti-Sm/RNP antibodies, together with longstanding photosensitivity, supported the diagnosis despite negative anti-double-stranded DNA antibodies, normal complement levels, and the absence of clinical evidence of systemic lupus erythematosus.

The clinical profile of our patient can also be compared with the retrospective series of 61 patients reported by Rangel et al. In that cohort, ulceration was observed in only 8% of patients, while buttock involvement accounted for 5.7% of affected sites. Moreover, 59% of patients had involvement of two or more anatomical regions, whereas our patient had a single lesion confined to the left buttock. These findings reinforce the unusual nature of the combined ulcerative and isolated gluteal presentation in our case [3].

Diagnostic delay has also been reported in lupus panniculitis. Rangel et al. found a mean delay of 19.2 months between symptom onset and diagnosis, increasing to 29.8 months in patients without pre-existing systemic or discoid lupus erythematosus. In our patient, the diagnosis was established after eight months of disease evolution. Although shorter than that reported in their cohort, this period was marked by several courses of systemic antibiotics, reflecting the diagnostic uncertainty commonly associated with atypical presentations [3].

The serological profile of our patient was also broadly consistent with the findings of Rangel et al. Antinuclear antibodies were detected in 75% of patients in their cohort, whereas anti-double-stranded DNA antibodies were less frequently positive. Our patient similarly had positive antinuclear antibodies but negative anti-double-stranded DNA antibodies. However, unlike a substantial proportion of patients in that series, our patient had no clinically apparent systemic or discoid lupus erythematosus at presentation [3].

Treatment of lupus panniculitis may require escalation beyond antimalarial therapy, particularly in persistent or corticosteroid-dependent disease [1,2,7]. Hydroxychloroquine and systemic corticosteroids produced only partial improvement in our patient, and repeated relapses occurred during corticosteroid tapering. Dapsone subsequently failed to achieve sustained disease control. Methotrexate was therefore added to ongoing corticosteroid therapy as a steroid-sparing agent and resulted in marked clinical improvement within three months, allowing further corticosteroid tapering. This treatment course is comparable to the therapeutic burden reported by Rangel et al., in whose cohort patients required a mean of 2.5 systemic therapies and 52% received at least one immunosuppressive agent. Methotrexate was among the systemic agents used in their series [3].

Residual atrophic scarring persisted despite clinical remission. Permanent lipoatrophy and contour changes are well-recognized sequelae of lupus panniculitis [1,3]. Similarly, Rangel et al. reported atrophy or contour changes in a substantial proportion of their patients, supporting the observation that structural damage may persist even after inflammatory activity has been controlled [3].

Taken together, our patient shared several features previously reported in lupus panniculitis, including pain, antinuclear antibody positivity, therapeutic refractoriness, and residual atrophy [1-3]. However, the association of ulceration with an isolated gluteal localization was distinctly uncommon compared with the Rangel et al. cohort [3].

This case report has some limitations. T-cell clonality analysis was not available at our institution, limiting additional assessment for a clonal T-cell lymphoproliferative process. In addition, apart from photographic documentation, clinical improvement was not formally assessed using standardized lesion measurements or quantitative symptom scores.

## Conclusions

This case underscores the diagnostic complexity of ulcerative lupus panniculitis, particularly when it presents as an isolated chronic gluteal ulcer. The unusual clinical presentation may mimic a wide range of infectious, inflammatory, and malignant conditions, resulting in considerable diagnostic delay. Recognition of suggestive clinical features, combined with timely histopathological examination and immunological assessment, is essential for establishing the diagnosis. In our patient, methotrexate was associated with sustained clinical improvement after failure of conventional therapy, suggesting that it may represent a useful steroid-sparing option in selected refractory cases. However, further studies are needed to better establish its efficacy in refractory lupus panniculitis. Awareness of this rare presentation may facilitate earlier diagnosis and improve patient outcomes.

## References

[1] Fraga J, García-Díez A (2008). Lupus erythematosus panniculitis. Dermatol Clin.

[2] Arai S, Katsuoka K (2009). Clinical entity of lupus erythematosus panniculitis/lupus erythematosus profundus. Autoimmun Rev.

[3] Rangel LK, Villa-Ruiz C, Lo K, Cobos G, Lo Sicco K, Vleugels RA, Femia AN (2020). Clinical characteristics of lupus erythematosus panniculitis/profundus: a retrospective review of 61 patients. JAMA Dermatol.

[4] Ng PP, Tan SH, Tan T (2002). Lupus erythematosus panniculitis: a clinicopathologic study. Int J Dermatol.

[5] Massone C, Kodama K, Salmhofer W (2005). Lupus erythematosus panniculitis (lupus profundus): clinical, histopathological, and molecular analysis of nine cases. J Cutan Pathol.

[6] Maverakis E, Ma C, Shinkai K (2018). Diagnostic criteria of ulcerative pyoderma gangrenosum: a Delphi consensus of international experts. JAMA Dermatol.

[7] Chasset F, Teboul A (2026). Update on cutaneous lupus erythematosus pathogenesis, diagnosis and management. J Eur Acad Dermatol Venereol.

